# Sudden Infant Death Syndrome: Beyond Risk Factors

**DOI:** 10.3390/life11030184

**Published:** 2021-02-26

**Authors:** Serafina Perrone, Chiara Lembo, Sabrina Moretti, Giovanni Prezioso, Giuseppe Buonocore, Giorgia Toscani, Francesca Marinelli, Francesco Nonnis-Marzano, Susanna Esposito

**Affiliations:** 1 Department of Medicine and Surgery, University Hospital of Parma, 43126 Parma, Italy; smoretti@ao.pr.it (S.M.); gprezioso@ao.pr.it (G.P.); giorgia.toscani@studenti.unipr.it (G.T.); francesca.marinelli@studenti.unipr.it (F.M.); susannamariaroberta.esposito@unipr.it (S.E.); 2 Department of Molecular and Developmental Medicine, University of Siena, 53100 Siena, Italy; Chiara.lembo@student.unisi.it (C.L.); giuseppe.buonocore@unisi.it (G.B.); 3 Department of Chemistry, Life Sciences and Environmental Sustainability, University of Parma, 43126 Parma, Italy; francesco.nonnismarzano@unipr.it

**Keywords:** SIDS, newborn infant, genetic polymorphism, neurotransmitter

## Abstract

Sudden infant death syndrome (SIDS) is defined as “the sudden death of an infant under 1 year of age which remains unexplained after thorough investigation including a complete autopsy, death scene investigation, and detailed clinical and pathological review”. A significant decrease of SIDS deaths occurred in the last decades in most countries after the beginning of national campaigns, mainly as a consequence of the implementation of risk reduction action mostly concentrating on the improvement of sleep conditions. Nevertheless, infant mortality from SIDS still remains unacceptably high. There is an urgent need to get insight into previously unexplored aspects of the brain system with a special focus on high-risk groups. SIDS pathogenesis is associated with a multifactorial condition that comprehends genetic, environmental and sociocultural factors. Effective prevention of SIDS requires multiple interventions from different fields. Developing brain susceptibility, intrinsic vulnerability and early identification of infants with high risk of SIDS represents a challenge. Progress in SIDS research appears to be fundamental to the ultimate aim of eradicating SIDS deaths. A complex model that combines different risk factor data from biomarkers and omic analysis may represent a tool to identify a SIDS risk profile in newborn settings. If high risk is detected, the infant may be referred for further investigations and follow ups. This review aims to illustrate the most recent discoveries from different fields, analyzing the neuroanatomical, genetic, metabolic, proteomic, environmental and sociocultural aspects related to SIDS.

## 1. Definition and Epidemiology

Sudden infant death syndrome (SIDS) is defined as “the sudden death of an infant under 1 year of age which remains unexplained after thorough investigation including a complete autopsy, death scene investigation, and detailed clinical and pathological review” [1,2]. SIDS is characterized by an unexpected death during the sleeping period and it typically occurs in the first 12 months of age in a previously healthy infant. Most events take place in the child’s home; the out-of-home deaths are most frequent at a relative’s home or a child-care setting, especially if the child sleeps in prone position and in a stroller and/or car seat [3]. SIDS is a subcategory of Sudden Unexpected Infant Deaths (SUID) and represents nearly half of these cases; SUID includes SIDS of unknown cause and also events of strangulation in bed or accidental suffocation [4]. The peak of incidence of SIDS is around two to four months of age and 90% of cases occur before six months of age; prevalence of SIDS is higher in boys than girls, at a 3:2 ratio [5].

In the late 1980s prone sleep has been documented as a major risk factor for SIDS, leading in the 1990s to “back-to-sleep” campaigns which have had a great impact on reduction of SIDS rates [6]. SIDS infant mortality has decreased well over 50% for most countries, especially in the first few years after the beginning of national campaigns [7]. Alongside the SIDS prevention recommendations, perinatal care has experienced numerous other improvements, so it’s difficult to attribute the SIDS incidence drop to only the supine sleep practice [6]. Furthermore, the increasing rates of other causes of death such as strangulation in bed and accidental suffocation may represent another explanation of the decline in SIDS rates [8]. Over the first decade of this century, infant mortality from SIDS has experienced a continuous drop in some countries such as Australia, Canada, England and Wales, Germany, Japan and the Netherlands, while it has remained stationary in others, notably the USA and New Zealand [9]. Despite continuous public health efforts concentrating on the improvement of sleep conditions with a special focus on the high-risk groups [10,11], incidence of SIDS still continues to be high. In fact, SIDS still represents a prominent cause of infant death, occurring at a rate of 27/100,000 live births in the United Kingdom and 38/100,000 the United States [12,13]. In Italy incidence is about 1 out of 1000 live births [14]. Therefore, continuous research on the cause and prevention of SIDS is needed.

## 2. Pathogenesis and Risk Factors 

The most recent evidence suggests that SIDS pathogenesis is a multifactorial condition that comprehends genetic, environmental and sociocultural factors [8]. The Triple-Risk Model, first described in 1994, affirms that SIDS occurs in infants with latent biological vulnerability (brainstem abnormality or genetic pattern), who is exposed to a trigger event or extrinsic risk factor (prone sleeping, airway obstruction) during a critical phase of development [15]. The combination of intrinsic and extrinsic factors which overlap during a period of respiratory, autonomic and cardiac development, usually occurring between two to four months of age, leads to a life-threatening event during a period of sleep. Failure of protective mechanisms during these episodes finally concludes with unexpected death. On the contrary, SIDS is less likely to occur with the removal of one of these factors [16,17]. 

Multiple extrinsic risk factors for SIDS in the sleep environment of the infants have been examined. Prone sleeping appears to be the most significant risk factor for SIDS. In fact, it is likely to be associated with re-breathing expired gases, suffocation, overheating and decreased arousal [18]. Infants in side lying position are also at high risk to roll into prone position while sleeping [19]. Other factors related to the sleeping environment involve soft bedding, sleeping with blankets, pillows, soft objects, bumper pads, head or face covered during sleep, bedsharing (especially co-sleeping on a couch or sofa) and room or infant overheating [20]. Maternal smoking during pregnancy is associated with a fivefold increase in SIDS events, and postnatal smoking exposure further increases the risk [21,22,23]. In fact, it’s been demonstrated that smoking during pregnancy contributes to the risk due to the disruption of arousal patterns in the sleeping baby along with impairment of autonomic system and cardiovascular response: uterine exposure reduces lung compliance and volume, alters arousal mechanisms and decreases heart rate variability in response to stress, all factors that can negatively affect a baby’s ability to respond appropriately to the environment [24,25]. A recent study showed a linear correlation between number of cigarettes smoked daily during pregnancy and the risk of a SUID event. Furthermore, quitting smoking over the three trimesters is strongly associated with great reduction of SUID risk but also diminution of cigarettes smoked daily contributes to a small decrease in risk [26]. Additional risk factors include maternal alcohol use, young maternal age (under 20 years) and poor prenatal care [27]. The combined exposure of alcohol and tobacco beyond the first trimester of pregnancy appears to have a synergistic effect on the risk for SIDS events [28]. Co-sleeping associated with recent use of alcohol or drugs by the parents also increases the risk significantly [29]. The strong association between smoking, alcohol consumption and drugs utilization may also explain, in part, the interaction described between co-sleeping and smoking [29,30,31,32]. Bottle-feeding is associated with increased risk, while the use of a pacifier and breastfeeding appear to be protective factors [27,33,34]. Some studies suggest that a significant part of SIDS cases may be closely related to sub-clinical infection processes [35,36]. In a study conducted by Goldwater et al. in 2020, significantly heavier thymus and brain were found in SIDS victims compared to non-SIDS controls. This finding is related to immune responses in the brain and thymus associated with possible subclinical infections [37].

Intrinsic risk factors are male gender, population subgroups such as non-Hispanic black infants, American Indian or Alaska Native infants [8,38] and prematurity. Preterm birth or low birth weight increases the risk of SIDS events three to four times, suggesting that altered intrauterine environment may contribute to the pathogenesis [39]. Additionally, smoking during pregnancy increases the risk of premature delivery [40]. Furthermore, research suggests that disorders of homeostasis, neuroregulation and cardiorespiratory function associated with brain and brainstem anomalies play an important role in SIDS [16]. In particular, serotonin brainstem abnormalities have been identified in up to 70% of infants who have died of SIDS [16]. Since the serotonin system is associated with several homeostatic functions, these anomalies may possibly lead to a network dysfunction that affects arousal and cardiorespiratory functions [41,42]. In addition to brain vulnerabilities, research has focused on identifying genetic variants related to defects involving autonomic and metabolic functions, neurotransmission and cardiac repolarization, suggested to contribute to SIDS infant’s “underlying vulnerability” [2,43,44]. Thus, certain infants may have a genetic predisposition to SIDS or an underlying abnormality in the brainstem, which becomes manifest when the infant experiences environmental challenges (hypoxia, asphyxia, hypercarbia, overheating) during sleep and differentiates a SIDS infant from a healthy infant. In fact, infants without the underlying conditions present an efficient protective brainstem response to homeostatic challenges which promptly manage to avoid SIDS occurrence [2]. The combination of multiple extrinsic and intrinsic factors leads to asphyxia. Vulnerable infants are not able to respond with arousal that prevents re-breathing or apnea. Consequently, asphyxia leads to bradycardia and insufficient gasping breathing, which eventually terminate with death [45].

Most of these life-threatening events occur during the sleep period. In fact, sleep is associated with a reduction of blood pressure, heart rate, respiratory rate and muscle tone, especially in the upper airways. During sleep phases protective reflexes to hypoxia and hypercapnia are also depressed. Blood pressure, cerebral oxygenation and cerebral vascular homeostasis are decreased in the prone position [46,47]. In fact, term infants between two to four months of age show a depressed baroreflex response and decreased arousal in the prone position [48,49]. In premature babies these characteristics in prone position are mostly marked [50,51]. These considerations highlight, indeed, the increased risk of SIDS occurring during sleeping, mainly in the prone position during specific infant development windows.

## 3. The Brainstem Hypothesis in SIDS

Neural research in SIDS has concentrated efforts to define the existence and eventual location of a pathological lesion in the brain. No major anatomic signs of neural pathology have been revealed by standard autopsy.

Impairment of the brainstem has been related to sudden death. The brainstem is the primary anatomic site of homeostatic control and sleep/waking regulation in the brain [52]. The brainstem hypothesis in SIDS suggests that developmental abnormalities in specific brainstem regions lead to a failure of protective mechanisms against exogenous stressors associated with asphyxia, hypoxia, hypercapnia, or thermal imbalance during sleep. Defense functions, as central chemosensitivity to carbon dioxide (CO_2_) and peripheral and central chemosensitivity to oxygen (O_2_), activate an autoresuscitation mechanism with arousal accompanied by head lifting to avoid asphyxia. Defects in this system include impaired arousal, ineffective respiratory pattern, episodes of obstructive apnea during sleep and autonomic dysfunction [53,54].

Additional evidence of the possible brainstem role in SIDS was reported by Naeye et al. who described astrogliosis in the medullary reticular formation in 50% of SIDS cases. This scarring lesion was interpreted as the result of chronic alveolar hypoventilation and hypoxemia [55]. Further studies confirmed the presence of reactive gliosis in different brainstem regions such as inferior olivary nuclei of the brainstem [56,57]. Gliosis is thought to be a sign of brainstem function impairment, as a result of hypoxia and chronic underventilation [58]. Finding a gliosis marker in the brainstem in SIDS cases supported the validity of brainstem hypothesis [59]. 

Furthermore, an immature developmental pattern in the SIDS brainstem has also been reported, with brainstem neurons presenting an augmented dendritic spine number [60]. These findings have been recently supported by proteomic investigations that showed abnormalities related to neuronal/glial/axonal growth, metabolism and apoptosis in some brainstem areas such as raphe, hypoglossal nuclei and medullary pyramids. These considerations suggest that brainstem immaturity, as well as gliosis, may be involved in the abnormal central respiratory and arousal control [61].

Multiple neurotransmitter networks anomalies are thought to be responsible for the underlying vulnerability in SIDS infants. These defects involve different brainstem neurochemicals such as catecholamines, neuropeptides, acetylcholinergic metabolites, amines, aminoacids (primarily glutamate), growth factors and some cytokines [62]. A defective binding of peptide neurotransmitter substance P to its receptor neurokinin-1 has been described in nuclei involved in cardiorespiratory and autonomic control. In fact, a defect of medullary substance P network with cerebellar sites may result in failure to activate respiratory and motor responses. In particular, low levels of substance P have been found in the olivary nuclei that control head and neck movements. As a consequence, failure of the protective mechanism such as head lifting or tilting to escape hypoxia may occur in SIDS infants [63]. Another study highlighted the interactions of GABA neurons in the medulla oblongata with the medullary serotonergic system in the regulation of homeostasis [64]. Data suggest that deficits of GABAergic and serotoninergic systems cooperate to generate medullary dysfunction in SIDS [65]. SIDS was also associated with low levels of tryptophan hydroxylase enzyme, which is involved in serotonin synthesis, resulting in decreased serotonin production [66]. 

However, researches have been mostly focused on the role of the brainstem serotoninergic system. The brainstem serotonin network in the rostral medulla is involved in the activation of the protective respiratory and autonomic reactions in response to exogenous stressors during sleep. According to the serotonin brainstem hypothesis, a defect in the serotonin system leads to failure of the autoresuscitation and arousal reaction that ultimately causes SIDS. A study conducted by Kinney et al. identified a serotonergic impairment in the medullary reticular formation of the brainstem as a “core” lesion in SIDS [65]. The affected serotonin brainstem network is supposed to involve serotonin neurons interconnected among arcuate nucleus, paragigantocellularis lateralis, gigantocellularis, intermediate reticular zone and caudal raphe [65]. These regions have been identified using a quantitative autoradiography which has demonstrated a decreased serotonergic receptor binding in these nuclei in SIDS cases compared to controls [67]. The ascending serotoninergic arousal system is also implicated. In fact, the rostral reticular formation dysfunction is postulated to be transmitted to median raphe and dorsal raphe of the ascending serotonergic arousal system that results in failure of the metabolic challenge response, leading to death [65]. In particular, an alteration of Pet-1 expressing neurons located in the serotonergic raphe system has been related to the impaired recovery system. This suggests a role of Pet-1 neuron activity in the neonatal survival mechanism responding to hypoxia [68]. Furthermore, recent findings by Haynes et al. have described increased serum serotonin levels in SIDS cases compared to controls. Therefore, a high serum serotonin may be utilized as a biomarker in SIDS autopsies to distinguish deaths caused by serotonin-related anomalies [69].

As potential causes of serotonin brainstem disruption in SIDS, the role of maternal and pregnancy factors has been discussed. In fact, analysis of the placentas of newborns who subsequently died of SIDS suggests that an infant’s vulnerability originates in the gestational period. 

In fact, some maternal factors associated with fetal hypoxia, such as placenta vascular hypoperfusion, maternal anemia and cigarette smoking generate a suboptimal intrauterine environment. These maternal factors are hypothesized to be responsible, in part, for impaired brain development, particularly in the central serotonin system, as the basis of vulnerability in sudden postnatal death [70,71,72]. Furthermore, some gene polymorphisms of the promoter region of a serotonin transporter protein (“L” allele and “LL” genotype variants) are responsible for increased activity of the serotonin transporter protein, resulting in reduced concentration of serotonin. These genetic variants have been frequently found in SIDS victims [16,62,73,74]. Interestingly, the same variants were also found in additional predisposing conditions [75]. However, some studies reported no association between LL genotype or L allele and SIDS in Caucasians [76,77]. 

Yet, the role of this polymorphism in other ethnicities as a risk factor in SIDS still needs to be clarified [78]. Additional studies should be carried out to assess the role of population genetics influencing serotonin transporter protein alleles and genotypes distribution in different ethnicities [74]. Different ethnic groups sharing the same social conditions show different SIDS rates [79,80]. Recently, a nearly fivefold variation in high risk of SIDS has been found between ethnic groups in England and Wales [81]. Authors speculated that cultural differences play an important role in infant care rather than genetic factors. Further research into infant-care practices in low-risk ethnic groups might enable more effective prevention of SIDS in the general population. While there is some evidence of how genetic differences might influence susceptibility to certain factors (e.g., inflammatory responses) [82], the knowledge about the functional impact of these differences is still lacking. As a matter of fact, there is no evidence that genetic influences outside of social and environmental factors pose a risk for SIDS. 

Brainstem dysfunction is not limited to only one neurochemical network, such as the serotoninergic system. In fact, recent studies conducted by Hunt at al. have reported abnormal expression in nine different proteins within some brainstem nuclei, mainly the raphe nuclei and pyramids, that may relate to developmental and neurological cyto-architecture abnormalities in SIDS cases [61]. A study by Lavezzi et al. recently described low tyrosine hydroxylase expression in SIDS cases associated with a delayed development of Substantia Nigra pars compacta (SNpc). Moreover, nicotine absorption in the uterus was related to decreased neuron density in SN [83]. SNpc represents the major dopamine brain center with an important role in regulation of the sleep-arousal cycle [84]. Therefore, the deficit of dopaminergic neurons, alongside with smoking exposure, may explain SIDS occurrence in the awakening phase in a significant number of cases [83].

## 4. Metabolic Predisposition

Inborn errors of metabolism (IEM) have been described as possible causes of SIDS [85,86,87]. Metabolomics analysis enables characterization of metabolites produced by cells, tissues and microorganisms, their quantification and interpretation [88]. A study conducted by Graham et al. analyzed data of Nuclear Magnetic Resonance and Mass Spectrometry from the medulla oblongata of infants who died from SIDS. This analysis revealed that fatty acid metabolism was the principal metabolic pathway altered in the brain of infants with SIDS occurrence, thus fatty acids oxidation disorders may represent one of many causes of SIDS. Furthermore, this analysis identified one metabolite (octadecenoyl-L-carnitine) that could be potentially used as a diagnostic tool for early screening to detect infants at greatest risk of SIDS occurrence. Further studies should determine if the same diagnostic biomarkers identified in this study can also be found in blood samples [89].

A recent study conducted a postmortem analysis of short chain fatty acids (SCFAs) values in babies who experienced SIDS or death for causes not related to SIDS. It’s described that a SFCAs quantitative profile, involving isobutyric, butyric, hexanoic, valeric, and acetic acids, allows identification of the risk of SIDS [90]. 

## 5. Proteomics

Proteomic analysis has also been conducted with the aim to identify different expression of proteins in SIDS [61]. A recent study applied proteomic techniques to characterize changes in the proteome related to hypoxia, inflammation and apoptosis of SIDS compared to age-matched controls, analyzing heart, medulla tissues and blood samples. Results showed differentially regulated proteins, especially APOA1, GAPDH, S100B, zyxin and complement component C4A in SIDS cases as compared to the controls. All of them appeared up-regulated in SIDS except C4A, which was down-regulated. These findings suggest the role of these proteins as potentially diagnostic biomarkers for SIDS [91]. Using proteomics as a discovery tool, a study by Broadbelt et al. described a significant reduction levels of isoforms of the 14-3-3 protein family, associated with anomalies in TPH2 and serotonin levels and serotonin receptor (5-HT1A) binding in SIDS cases [92]. In fact, findings suggested that the deficit of 14-3-3, necessary for TPH2 modulation [93,94,95], may lead to TPH2 deficiency and consequently to medullary serotonin system impairment, resulting in SIDS [92].

## 6. Genetic Predisposition

Genetic studies have mostly focused on the ion channels of heart mutations. Polymorphism in sodium and potassium channel genes have been reported, including the sodium channel gene SCN5A, which is associated with prolonged QT intervals and may also be responsible for altered autonomic system development [96]. In fact, initial findings have showed that 2% of SIDS cases carried gain-of-function mutations in the sodium channel encoded by SCN5A gene [97]. Studies also described some inherited cardiac diseases such as Long and short QT syndrome, Brugada syndrome, catecholaminergic polymorphic ventricular Tachycardia and hypertrophic cardiomyopathy as monogenic causes in some SIDS cases [44,98,99,100,101,102]. It’s been suggested that long QT syndrome (LQTS) could explain 10% of all current SIDS cases [98,103]. 

With the development of next-generation sequencing (NGS), the analysis of the whole exome is expanding the identification of mutations and also potential pathogenic sequence variations responsible for underlying vulnerability in SIDS deaths. Many researches have described pathogenic mutations in genes associated with cardiac channelopathies responsible for SIDS occurrence in up to 30% of cases as monogenic cause of death [44,101,102]. In a study of variant analysis conducted by Tester et al., only 4.3% of the European SIDS cases possessed pathogenic variations in one of the 90 genetic heart disease genes analyzed [104]. Another recent study by Köffer et al. assessed the percentage of rare and ultrarare variants (respectively minor allele frequency ≤0.2% and ≤0.005%) in genetic heart disease genes. In only 6% of these cases, gene ultrarare variants were considered potentially pathogenic. Therefore, still rare variant interpretations associated with inherited cardiac diseases need to be standardized [105]. A recent study by Liebrechts-Akkerman et al. published in 2020 conducted an analysis using a targeted massively parallel sequencing exon screening, differently from other targeted genotyping SIDS studies. This analysis allowed detecting either novel or previously known exonic DNA variants in selected arrhythmia genes. These findings provided further evidence for cardiac arrhythmias as partial genetic explanation of SIDS. Thus, the authors stress the importance of standardized DNA testing for LQTS and other cardiac arrhythmia genes as an essential element in the ordinary SIDS diagnostic protocol. Furthermore, it’s proposed as an additional preventive measure to perform a standard ECG testing in newborns during the first two weeks of life [106]. Another recent study of 2020 by Simma et al. proposed performing standardized neonatal ECG screening in the first days of life with the aim of detecting neonates with a significant transient form of prolonged QT intervals and helping the diagnosis of congenital LQTS. This study suggests applying genetic testing as a second step in case of abnormal ECG results. Furthermore, in this study pathological cases were treated with a beta blocking agent for the first years of life [107]. 

Implementation of such a strategy would place considerable strain on the health care system. It might also result in increased financial costs and could influence parental behavior. Many questions remain opened: how many lives will ECG save? What do we know about ‘normal’ and ‘abnormal’ results that may influence what we do next? Will parents with a ‘normal’ result be less likely to follow safer sleep advice? Will parents with an ‘abnormal’ result become very anxious? In absence of clear answers, this topic needs to be carefully thought by planning much more research.

Other studies examined the potential role of noncardiac genes in the pathogenesis of SIDS. According to these studies, 61 genes were identified as potential “SIDS-susceptibility” genes, in the variant analysis typically in the promoter region. Yet, only around 55% of these genes have been involved on a monogenic basis in SIDS [108,109,110,111,112]. Of note, it has been described as a significant overrepresentation of functionally disruptive variants in the SCN4A gene. This gene encodes skeletal muscle voltage-gated sodium channel (Nav1.4), involved in the skeletal respiratory muscle contraction which have a key role in SIDS pathogenesis [113]. A recent study also reported SCN1A variants from exome sequencing in two infants who died of SIDS [114]. A study conducted by Gray et al. performed a SIDS-susceptibility variant analysis of these 61 previously published noncardiac SIDS-susceptibility genes. According to the findings of this research, there is very limited evidence that these specific genes are implicated in SIDS susceptibility in a monogenic basis with autosomal dominant and recessive inheritance. Therefore, it still needs to be investigated whether infant vulnerability to sudden death may be supported by a more complex polygenic inheritance model [115].

The involvement of additional genes regulating the metabolism of neurotransmitters (mainly serotonin and dopamine) has already been described in the previous chapter “The brainstem hypothesis in SIDS” [41,73,74,77]. 

Finally, genetic predisposition has also been related to increased vulnerability to smoke exposure. In fact, a recent study analyzed the contribution of GSTs (enzymes of glutathione-S-transferase supergene family), involved in detoxification of xenobiotics [116]. A significant correlation has been described between the GSTM1 deletion characterizing the gene polymorphism, resulting in a lack of metabolic activity, and SIDS exposed to smoking. These findings highlight the role of smoking exposure as an important SIDS risk factor connected with a biological predisposition [117].

## 7. Recommendations on Safe Infant Sleeping Environment 

In November 2016, the American Academy of Pediatrics task force published updated recommendations to reduce risk of SIDS and sleep-related infant deaths. These recommendations are addressed to infants up to one year of age. The strength of the guidelines is based on case-controlled studies as randomized trials cannot be performed for SIDS [16].

The sleeping position is the “strongest modifiable risk factor for SIDS” [16,32]. Infants should sleep in the supine position until one year of age or until the infant is able to roll from back to supine position, unassisted. Of note, supine position has not been related to increased risk of choking or aspiration, even in infants with gastroesophageal reflux disease [118]. Supine position is also recommended for premature infants either in NICU and home settings [40].

A firm flat surface covered only by a thin, fitted sheet should be used. Soft items such as toys, crib bumpers, positioners and pillows should be avoided, as well as loose sheets and blankets, due to risk of suffocation or airway obstruction [16]. Car seats, infants swings or strollers should not be used for routine sleep. The infant who fell asleep in such equipment should be repositioned to on an appropriate surface as soon as possible, due to the risk of head flexion resulting in obstruction of the upper airways [119]. The sleep surface must be located in a hazard-free location, without dangling cords or electric wires [16].

Overheating of infants by overwrapping, head coverings and excessive clothing must be avoided [120]. Room sharing is encouraged at least during the first six months of age, but bed sharing is prohibited until one year of age. In fact, sleeping in the caregiver’s bed, couch or chairs put the infant at risk of overheating, sleeping on a soft surface and being rolled over by adults. Conversely, room sharing has been shown to decrease SIDS risk by 50% [27].

Breastfeeding appeared to be one of the strongest protective measures against SIDS. Breastfeeding benefit is stronger when breastfeeding is exclusive, reducing the risk of SIDS by approximately 50% if conducted over the first month of life [121]. Furthermore, it was shown that any breastfeeding, exclusive or with formula supplementation, was able significantly protect against SIDS. In addition, the protective effect increases proportionally to the duration of breastfeeding [121].

The pacifier use for naps or bedtime is also considered a protective factor against SIDS. The exact protective mechanism is still unclear; it is assumed that the use of the pacifier may increase autonomic control and cardiovascular stability which help to maintain a patency of the airways. No adverse effect was found of breastfeeding with the use of the pacifier [33,34]. However, it is recommended to introduce pacifiers to infants only after breastfeeding is well established. The pacifier should not be attached to any strings or cords as these might present a risk of strangulation [33,34,122]. 

The routine use of home apnea monitors is not recommended in infants, including preterm and infants at risk of SIDS. In fact, cardiorespiratory monitors have not been demonstrated to decrease incidence of SIDS [123]. Furthermore, the usage of these tools may distract from adoption of other effective measures or give false alarms that could lead to overdiagnosis with consequential unnecessary analysis and caregiver anxiety [124].

Prenatal care should be endorsed from early pregnancy. Smoking, alcohol consumption and illicit drugs should be avoided by women during pregnancy, after delivery and during breastfeeding, as these factors significantly increase the risk of SIDS occurrence [16,22,23].

Regular immunization in accordance with Centers for Disease Control and Prevention schedule should be followed as it has been demonstrated to be protective and not associated with SIDS risk [16].

Prone position or “tummy time” is recommended only if supervised, when infants are awake and alert, as its benefits motor development and helps minimize positional plagiocephaly [125]. There is no evidence that suggests that swaddling used as a strategy to promote sleep and calm the infant reduces the risk of SIDS. When swaddling practice is performed, infants should be positioned on their back. In fact, recent studies described an increased risk of SIDS in case the swaddled infants are placed in or rolls to the prone position [126]. 

Finally, education by health care professionals and implementation of safe sleep practices should be based on the model of SIDS risk-reduction recommendations. A recent study described a functional use of smartphone technology for prevention, by assessing infant sleep safety practices among at-risk communities. It’s been observed that photographs processed by coders provide a cost and timesaving assessment that may support safe sleep interventions in clinical and community settings [127].

Ongoing research into the etiology of SIDS and other sleep-related infants’ deaths is encouraged to help achieve the ultimate goal of completely eradicating SIDS deaths [16].

## 8. Conclusions

SIDS occurrence is associated with multifactorial conditions. While extrinsic factors have been largely recognized and significantly reduced through recommendations on safe sleep worldwide, understanding the underlying intrinsic vulnerability to SIDS still represents a challenge.

Biomarkers such as proteins, metabolites and neurotransmitters have been proposed for early identification of cases at risk. To eliminate SIDS cases related to cardiac channelopaties, it has been also proposed that an ECG is performed on all neonates within two weeks of life with subsequent genetic analysis if some alterations are detected, but the implementation of such a strategy requires further research.

New “omic” technologies provide a large amount of data that can be analyzed independently and combined, allowing detection of multiple system alterations.

A complex model that combines different risk factors data from biomarkers and omic analysis may represent a tool to identify a SIDS risk profile in newborn settings. If high risk is detected, the infant may be soon referred for further investigations and follow up. 

Whole exome sequencing of newborns in NICUs is another promising area to reveal susceptibility to SIDS. [128]. This new technology opens the possibility of extending the concept of precision medicine to an early stage of life. 

## Data Availability

Data is contained within the article.

## Abbrevations

LQTS	Long QT syndrome
SIDS	Sudden Infant Death Syndrome
SNpc	Substantia Nigra pars compacta
SP	Substance P
SUID	Sudden Unexpected Infant Death
TPH	tryptophan hydroxylase gene
